# Beneficial Effects of Neurotrophin-4 Supplementation During *in vitro* Maturation of Porcine Cumulus-Oocyte Complexes and Subsequent Embryonic Development After Parthenogenetic Activation

**DOI:** 10.3389/fvets.2021.779298

**Published:** 2021-11-12

**Authors:** Mirae Kim, Seon-Ung Hwang, Junchul David Yoon, Joohyeong Lee, Eunhye Kim, Lian Cai, Gahye Kim, Hyerin Choi, Dongjin Oh, Sang-Hwan Hyun

**Affiliations:** ^1^Laboratory of Veterinary Embryology and Biotechnology (VETEMBIO), Veterinary Medical Center and College of Veterinary Medicine, Chungbuk National University, Cheongju, South Korea; ^2^Institute of Stem Cell & Regenerative Medicine (ISCRM), Chungbuk National University, Cheongju, South Korea; ^3^Graduate School of Veterinary Biosecurity and Protection, Chungbuk National University, Cheongju, South Korea

**Keywords:** neurotrophin-4, pig, follicular development, *in vitro* maturation of oocytes, epidermal growth factor

## Abstract

Neurotrophin-4 (NT-4) is a neurotrophic factor that plays an important role in follicular development and oocyte maturation. However, it is not yet known whether NT-4 is related to oocyte maturation and follicular development in pigs. This study aims to investigate the effects of NT-4 supplementation during *in vitro* maturation (IVM) of porcine oocytes and subsequent embryonic development after parthenogenetic activation (PA). First, NT-4 and its receptors (TrkB and p75^NTR^) were identified through fluorescent immunohistochemistry in porcine ovaries. NT-4 was mainly expressed in theca and granulosa cells; phospho-TrkB and total TrkB were expressed in theca cells, granulosa cells, and oocytes; p75^NTR^ was expressed in all follicular cells. During IVM, the defined maturation medium was supplemented with various concentrations of NT-4 (0, 1, 10, and 100 ng/mL). After IVM, the nuclear maturation rate was significantly higher in the 10 and 100 ng/mL NT-4 treated groups than in the control. There was no significant difference in the intracellular reactive oxygen species levels in any group after IVM, but the 1 and 10 ng/mL NT-4 treatment groups showed a significant increase in the intracellular glutathione levels compared to the control. In matured cumulus cells, the 10 ng/mL NT-4 treatment group showed significantly increased cumulus expansion-related genes and epidermal growth factor (EGF) signaling pathway-related genes. In matured oocytes, the 10 ng/mL treatment group showed significantly increased expression of cell proliferation-related genes, antioxidant-related genes, and EGF signaling pathway-related genes. We also investigated the subsequent embryonic developmental competence of PA embryos. After PA, the cleavage rates significantly increased in the 10 and 100 ng/mL NT-4 treatment groups. Although there was no significant difference in the total cell number of blastocysts, only the 10 ng/mL NT-4 treatment group showed a higher blastocyst formation rate than the control group. Our findings suggest that supplementation with the 10 ng/mL NT-4 can enhance porcine oocyte maturation by interacting with the EGF receptor signaling pathway. In addition, we demonstrated for the first time that NT-4 is not only required for porcine follicular development, but also has beneficial effects on oocyte maturation and developmental competence of PA embryos.

## Introduction

Reproductive biotechnology plays an essential role in understanding the process of transferring genetic information through germ cells to the next generation (1, 2). One of the popular reproductive biotechnology techniques, *in vitro* embryo production, has great potential to generate transgenic animals and to better control the reproduction of livestock, such as pigs and cows (3, 4). Pigs are especially useful models for xenotransplantation and translational medicine research because they have genetic, anatomical, and physiological characteristics similar to those of humans (5, 6). In recent decades, many researchers have attempted to improve the efficiency of *in vitro* production of porcine embryos, including oocyte maturation (7–10). Ovarian folliculogenesis occurs under the influence of intra-ovarian factors (11) such as epidermal growth factor (EGF), fibroblast growth factor, and vesicular endothelial growth factor *in vivo*; however, these factors are not sufficient in the process of *in vitro* maturation (IVM) of oocytes, which may lead to incomplete cytoplasmic maturation and decreased IVM efficiency (12–14).

Neurotrophins are neurotrophic factors that are mainly involved in the survival and differentiation of neurons in the central and peripheral nervous systems (15, 16). Neurotrophins affect not only neural development, but also the development of the ovary, which is a highly innervated tissue (17–19). The mammalian neurotrophin family consists of nerve growth factor (NGF), brain-derived neurotrophic factor (BDNF), neurotrophin-3 (NT-3), and neurotrophin-4 (NT-4, also known as NT-4/5 or NT-5) (20). Among these neurotrophins, NT-4 is a member of the NGF family and an intra-ovarian follicular factor related to the transforming growth factor β superfamily (21). Thus, it is an important growth factor that plays a supporting role in ovarian follicular development and oocyte maturation through its interaction with the high-affinity tropomyosin receptor kinase B (TrkB) and the low-affinity pan-neurotrophin receptor (p75^NTR^) (22–24). Both Nt-4 and TrkB mRNAs are expressed in the neonatal rat ovary, and NT-4 can affect early follicular development in rats (17). BDNF, which binds to TrkB like NT-4, has already been shown to improve mammalian oocyte maturation (25–27), but there has been limited research on the relationship between NT-4 and porcine oocyte maturation.

Ovarian follicle development is an essential process in female reproduction. After primordial germ cells migrate to the genital ridge, they differentiate into oogonia in the putative ovary. In humans, the oogonia divide by extensive mitosis and become primary oocytes before birth. The primordial follicular assembly (primordial follicle formation) occurs at this stage of the differentiation from oogonia to primary oocytes. Therefore, primordial follicle assembly is very important for the acquisition of fertility in mammalian female reproduction (28). Numerous studies have shown that NT-4 promotes follicular assembly in rodents and humans (17, 18, 29–31). NT-4 mRNA and protein are expressed in oocytes and pre-granulosa cells present in the human pre-antral follicle stage (31). Furthermore, NT-4 improves human follicular assembly *in vitro* (32). NT-4, which has similar roles to BDNF in ovarian follicles, also promotes nuclear maturation and first polar body extrusion in mouse oocytes (21). Therefore, NT-4 has a beneficial effect on oocyte maturation in rodents, but it is unknown how it affects oocyte maturation in pigs.

The interaction between EGF-like peptides (e.g., amphiregulin, epiregulin, betacellulin, neuregulin 1–4, and transforming growth factor-α) and epidermal growth factor receptor (EGFR, also known as ERBB1) is known to be closely related to cell proliferation, survival, adhesion, and invasion (33). The EGFR signaling pathway, which is the most well-known receptor tyrosine kinase (RTK), can activate the MAPK, Akt-PI3K, and PLC-γ1-PKC pathways. In female reproduction, the EGFR signaling pathway is required for oocyte maturation, ovulation, and steroid production (34). RTKs such as EGFR and Trk family play critical roles in cellular response, cell proliferation, differentiation, and survival (35). Previous studies have shown that both EGF and neurotrophic factors such as BDNF (25) or glial cell line-derived neurotrophic factor (GDNF) (36, 37) may improve oocyte maturation and embryonic developmental potential in pigs. Although these studies have demonstrated the physiological roles of neurotrophic factors (BDNF or GDNF) in promoting follicle development or oocyte maturation in pigs, but there were no studies analyzing the specific gene expression mechanisms involved in the combined effects of EGF and NT-4. Therefore, we aimed to identify the physiological roles of NT-4 in porcine follicular development and oocyte maturation and to analyze specific mechanisms related to interaction of the EGFR signaling pathway in cumulus-oocyte complexes (COCs).

Here, we investigated the effects of NT-4, a neurotrophic factor, on IVM of porcine COCs and subsequent embryonic developmental competence after parthenogenetic activation (PA). We also identified NT-4 and its receptors (TrkB and p75^NTR^) in porcine ovaries using fluorescent immunohistochemistry (IHC). Finally, we studied the combined effect of NT-4 and EGF during IVM of porcine COCs.

## Materials and Methods

### Chemicals and Reagents

Recombinant human NT-4 (450-04) was purchased from Peprotech (Rocky Hill, NJ, USA). It was dissolved in Dulbecco's phosphate-buffered saline (dPBS; LB 001-02, WELGENE, Gyeongsan, Gyeongsangbuk-do, Republic of Korea) containing 0.1% (w/v) bovine serum albumin (A6003-25G). The working solution was stored at −70°C until use in the experiment. Unless otherwise noted, all chemicals and reagents used in this study were purchased from Sigma-Aldrich Corporation (St. Louis, MO, USA).

### Oocytes Collection and *in vitro* Maturation (IVM) of Porcine Cumulus-Oocyte Complexes (COCs)

Porcine ovaries were collected from a local slaughterhouse and placed in a thermos containing 0.9% (w/v) NaCl saline, transferred to the laboratory within 3 h, and maintained at a temperature of 37–39°C. After washing the ovaries with 0.9% NaCl saline two or three times, porcine follicular fluid (pFF) containing COCs was aspirated from the 3–6 mm ovarian follicles using a syringe with an 18 G needle. The aspirated pFF was placed in a 15 mL tube and inserted into a heat block to maintain the temperature at 37–39°C. After 5 min of follicular fluid contents sedimentation, the supernatant was removed and the washing process was repeated twice with HEPES-buffered Tyrode's medium containing 0.05% (w/v) polyvinyl alcohol (TLH-PVA). COCs with three or more compact cumulus cell (CC) layers and a homogeneous cytoplasm were observed and selected under a stereomicroscope (SZX-ILLK100, Olympus Optical Co., LTD., Tokyo, Japan). After adding 500 μL of maturation medium to each well in a Nunc 4-well dish (Nunc, Roskilde, Denmark), ~50–60 COCs were transferred to each well. The composition of the maturation medium was as follows: 0.6 mM cysteine, 0.91 mM sodium pyruvate, 10 ng/mL EGF, 75 μg/mL kanamycin, 1 μg/mL insulin, and 0.1% (wt/vol) PVA added to the TCM199 medium (Gibco, Grand Island, NY, USA). During the entire IVM period, NT-4 was added to the maturation medium at concentrations of 0, 1, 10, and 100 ng/mL. In the first 22 h of IVM, COCs were cultured in the presence of 10 IU/mL equine chronic gonadotropin (eCG) and 10 IU/mL human chronic gonadotropin (hCG) in the maturation medium, and in the next 20 h of IVM, COCs were cultured in the absence of eCG and hCG in the maturation medium (38). All IVM procedures were performed in a 39°C humid incubator with 5% CO_2_. The COCs that were matured for a total of 42 h were transferred to the TLH-PVA medium, and 0.1% hyaluronidase was added to COCs for denuding. Matured oocytes and CCs isolated from COCs were used in the subsequent experiments.

### Detection of NT-4 and Its Receptors in Porcine Intra-Ovarian Follicular Cells via Reverse Transcription Polymerase Chain Reaction (RT-PCR) Analysis

RT-PCR analysis was performed to understand whether NT-4 and its receptors (TrkB and p75^NTR^) are expressed in porcine intra-ovarian follicular cells. First, mural granulosa cells (GCs) and immature oocytes were isolated from pFF, and metaphase II (MII) oocytes (matured oocytes) and matured CCs were obtained after 42 h of IVM. RNA extraction was performed using TRIzol reagent (TaKaRa Bio, Inc., Otsu, Shiga, Japan), and complementary DNA (cDNA) synthesis was carried out using a reverse transcription master mix (Elpis Bio, Inc., Chungcheongnam-do, Daejeon, Republic of Korea) according to the manufacturer's instructions. Next, RT-PCR analysis was performed using 1 μL of cDNA template with 10 pmol of specific forward and reverse primers (Macrogen, Inc., Seoul, Republic of Korea), two units of Taq polymerase (Elpis Bio), 2 μL of 10× PCR buffer (Elpis Bio), and 5 pmol of dNTP mix (iNtRON Biotechnology, SungNam, Republic of Korea). PCR amplifications were conducted for 30 cycles with the following steps: denaturation at 95°C for 30 s, annealing at 57°C for 30 s, and extension at 72°C for 30 s. All primer sequences are listed in Supplementary Table 1. Each mRNA expression value was normalized to that of *18S ribosomal RNA* (*RN18S*). The PCR products were analyzed on a 0.9% agarose gel pre-stained with RedSafe Nucleic Acid Staining Solution (iNtRON Biotechnology).

### Fluorescent Immunohistochemistry of Paraffin-Embedded Ovarian Sections

Fluorescence IHC was performed as described previously (38). First, the porcine ovaries obtained from the slaughterhouse were washed with dPBS. To prepare paraffin-embedded tissue slides, porcine ovaries with small (1–2 mm), medium (3–6 mm), and large (7–9 mm) follicles were fixed in 10% formalin for 48 h at room temperature (RT). Then, tissues sliced to a thickness of 3 mm or less were placed in a tissue cassette and rinsed with running tap water for at least 10 min. The process of preparing paraffin blocks and paraffin section slides through dehydration and cutting was commissioned by the Laboratory Animal Center of Chungbuk National University (Cheongju, Chungcheongbuk-do, Republic of Korea). The paraffin section slide was observed under a microscope, and only the best tissue slides were selected and stored at 4°C until use.

Before deparaffinization, the tissue slide was placed in a dry oven at 60°C for ~30 min to remove moisture. For deparaffinization, the process of soaking in xylene for 5 min was repeated twice. The paraffin-free tissue slides were rehydrated by washing with 100, 90, 80, and 70% ethanol for 5 min each. The rehydrated tissue slides were rinsed under running tap water for 10 min, placed in 10 mM sodium citrate buffer (pH 6.0) (pre-heated to 100°C), and heated for 20 min to retrieve the antigen. After antigen retrieval by heating, the slides were cooled at RT for at least 30 min. The cooled slides were washed twice with Tris-buffered saline (TBS-T, pH 7.4) for 5 min each. Prior to the blocking step, the slides were drained and a wide border was drawn around the tissue using a hydrophobic pen (ImmEdge™ hydrophobic barrier PAP pen; Vector Laboratories, Inc., Burlingame, CA, USA). Next, the slides were placed in a humid chamber. Then, blocking buffer (10% goat serum in dPBS) was added to each tissue slide and incubated at RT for 2 h. After washing three times with TBS-T for 5 min at RT, each slide was treated with a primary antibody (diluted in blocking buffer) and incubated in a humid chamber overnight at 4°C. The antibody lists used in this study are summarized in Supplementary Table 2.

The next day, the slides were washed three times for 5 min each with TBS-T to remove the primary antibody solution, and then the secondary antibody solution (diluted in TBS-T) was added and incubated at RT for 1 h. The slides were washed again three times for 5 min at RT with TBS-T, and the nuclei were counterstained with 10 μg/mL Hoechst 33342. Finally, after mounting each slide using an anti-fade mounting solution (Molecular Probes, Inc., Eugene, OR, USA), the ovarian tissue sections were examined using a confocal laser microscope (Carl Zeiss, Thornwood, NY, USA), and all images were analyzed using the ZEN (blue edition) software program (38).

### Evaluation of Nuclear Maturation Using Fluorescent Staining

The rate of nuclear maturation was assessed after a total of 42 h of IVM. The matured COCs were mechanically denuded of CCs in the TLH-PVA medium with 0.1% hyaluronidase. The matured oocytes were fixed in dPBS containing 4% paraformaldehyde for 5 min and stained with TLH-PVA containing 10 μg/mL Hoechst 33342 for 5 min. The stained oocytes were examined using a fluorescence microscope (TE300, Nikon, Tokyo, Japan) and classified into four stages: germinal vesicle, metaphaseI, anaphase-telophase I, and MII according to nuclear maturation.

### Assessment of Cumulus Cell Expansion Index

The cumulus cell expansion index was estimated using a previously reported scoring system (0–4) (39). Briefly, a score of 0 indicates no expansion, a score of 1 indicates minimal expansion of the outermost CC layers, 2 indicates expansion of only the outermost CC layers, 3 indicates expansion of all CC layers except the corona radiata, and 4 indicates expansion of all CC layers including the corona radiata.

### Measurement of Intracellular Glutathione and Reactive Oxygen Species Levels

After 42 h of IVM, intracellular glutathione (GSH) and reactive oxygen species (ROS) levels in MII oocytes of each group were measured using a previously described method (40). In brief, CellTracker Blue 4-chloromethyl-6,8-difluoro-7-hydroxycoumarin (CMF2HC; Invitrogen) was used to detect GSH in the cytoplasm, and 2′,7′-dichlorodihydrofluorescein diacetate (H_2_DCFDA; Invitrogen Corporation, Carlsbad, CA, USA) was used to detect intracellular ROS. Ten oocytes from each group were incubated in the TLH-PVA medium, which contains 10 μM CMF2HC or 10 μM H_2_DCFDA, and stained in the dark for 30 min. The stained oocytes were carefully washed thrice in TLH-PVA and transferred to 10 μL droplets of TLH-PVA. Fluorescence measurements were performed using a fluorescence microscope (TE300, Nikon) with ultraviolet (UV) filters (370 nm for GSH and 460 nm for ROS). The fluorescence intensity of each oocyte was analyzed using Adobe Photoshop CS3 Extended program (version 10, San Jose, USA) and normalized to that of the control group.

### Quantitative Reverse Transcription Polymerase Chain Reaction (qRT-PCR) for Relative Gene Expression Analysis

After IVM was completed, 0.1% hyaluronidase was added to 50–60 COCs in each group, and matured oocytes and CCs were separated and sampled, respectively. RNA extraction and cDNA synthesis were conducted as described in the detection of NT-4 and its receptors in porcine intra-ovarian follicular cells via reverse transcription polymerase chain reaction (RT-PCR) analysis. To perform qRT-PCR, synthesized cDNA (1.2 μg/μL for CCs, 0.5 μg/μL for oocytes), 2× SYBR Premix Ex Taq (TaKaRa Bio, Inc.), and 10 pmol of specific primers (Macrogen) were prepared to make PCR mixtures. All primer sequences used in this experiment are listed in Supplementary Table 3. The qRT-PCR analysis was performed using the CFX96 Touch Real-Time PCR Detection System (Bio-Rad, Hercules, CA, USA). The reactions were performed for 40 cycles of denaturation at 95°C for 30 s, annealing at 57°C for 30 s, and extension at 72°C for 30 s. Relative quantification was performed using threshold cycle (Ct)-based methodologies at constant fluorescence intensity. The relative mRNA expression (R) was calculated using the following equation: *R* = 2^−[ΔCt sample−ΔCt control]^ (41). The expression of each mRNA was normalized to that of *glyceraldehyde 3-phosphate dehydrogenase* (*GAPDH*) for CCs and *RN18S* for oocytes.

### Parthenogenetic Activation and *in vitro* Culture (IVC) of Porcine Embryos

After IVM, the MII oocytes of each group were denuded as mentioned above (in the oocyte collection and *in vitro* maturation (IVM) of porcine cumulus-oocyte complexes (COCs) section) and washed in calcium-free TLH-PVA medium. Parthenogenetic activation was performed using previously described methods (40, 42). For activation, the MII oocytes were rinsed in 280 mM mannitol solution, which contains 0.01 mM CaCl_2_ and 0.05 mM MgCl_2_. Then, the matured oocytes were loaded into the activation medium (260 mM mannitol solution supplemented with 0.001 mM CaCl_2_ and 0.05 mM MgCl_2_) between the electrodes of the chamber. The chamber was connected to an electrical pulsing machine (LF101; Nepa Gene, Chiba, Japan), and the matured oocytes were activated under two direct-current (DC) pulses of 120 V/mm for 60 μs. The electrically activated oocytes from each group were immediately transferred into an IVC medium (porcine zygote medium; PZM-3) (43), which contains 5 μg/mL cytochalasin B and incubated in a 39°C humidified atmosphere of 5% CO_2_ and 95% N_2_ for 4 h. After 4 h, the cytochalasin B-treated oocytes were washed twice in a fresh IVC medium and transferred into droplets of 30 μL of the fresh IVC medium (10 activated oocytes per drop) covered with mineral oil. The PA embryos were cultured at 39°C in a humid incubator with 5% O_2_, 5% CO_2_, and 90% N_2_ for 7 d. The IVC medium was renewed in fresh 30 μL of PZM-3 droplets after 48 h and 96 h.

### Embryo Quality Evaluation and Total Cell Counts

The cleavage of PA embryos was evaluated on day 2. The normally cleaved embryos were assigned to one of three groups (only 2- to 3-cell, 4- to 5-cell, and 6- to 8-cell stage embryos, except for the 1-cell stage and fragmented embryos). On day 7, blastocyst formation was classified into three groups (early, expanded, and hatched blastocyst). To check the blastocyst quality, all blastocysts of each group were collected and washed twice in the TLH-PVA medium. Following the final wash, the blastocysts were fixed in 3.7% paraformaldehyde with PBS-PVA for 5 min and rinsed in PBS-PVA. The fixed blastocysts were stained with 10 μg/mL Hoechst-33342 for 5 min, mounted on glass slides in 100% glycerol droplets, and gently covered with a coverslip. Finally, they were observed using a fluorescence microscope (TE300, Nikon) with a UV filter (370 nm) and counted manually.

### Statistical Analysis

All experiments were conducted at least three times. Statistical analyses were carried out using IBM SPSS Statistics software (version 21.0; IBM Corp., Armonk, NY, USA). The rates of nuclear maturation, the levels of intracellular GSH and ROS, embryonic development data (e.g., the rate of cleavage and blastocyst formation, and total cell number of blastocysts), and the relative gene expression levels were compared via one-way analysis of variance (ANOVA), followed by Duncan's multiple range test. All data are reported as mean ± standard error of the mean (SEM). Statistically significant differences were considered when *p* < 0.05.

## Results

### Identification and Localization of NT-4 and Its Receptors in Porcine Ovarian Follicular Cells

**Figure 1:**
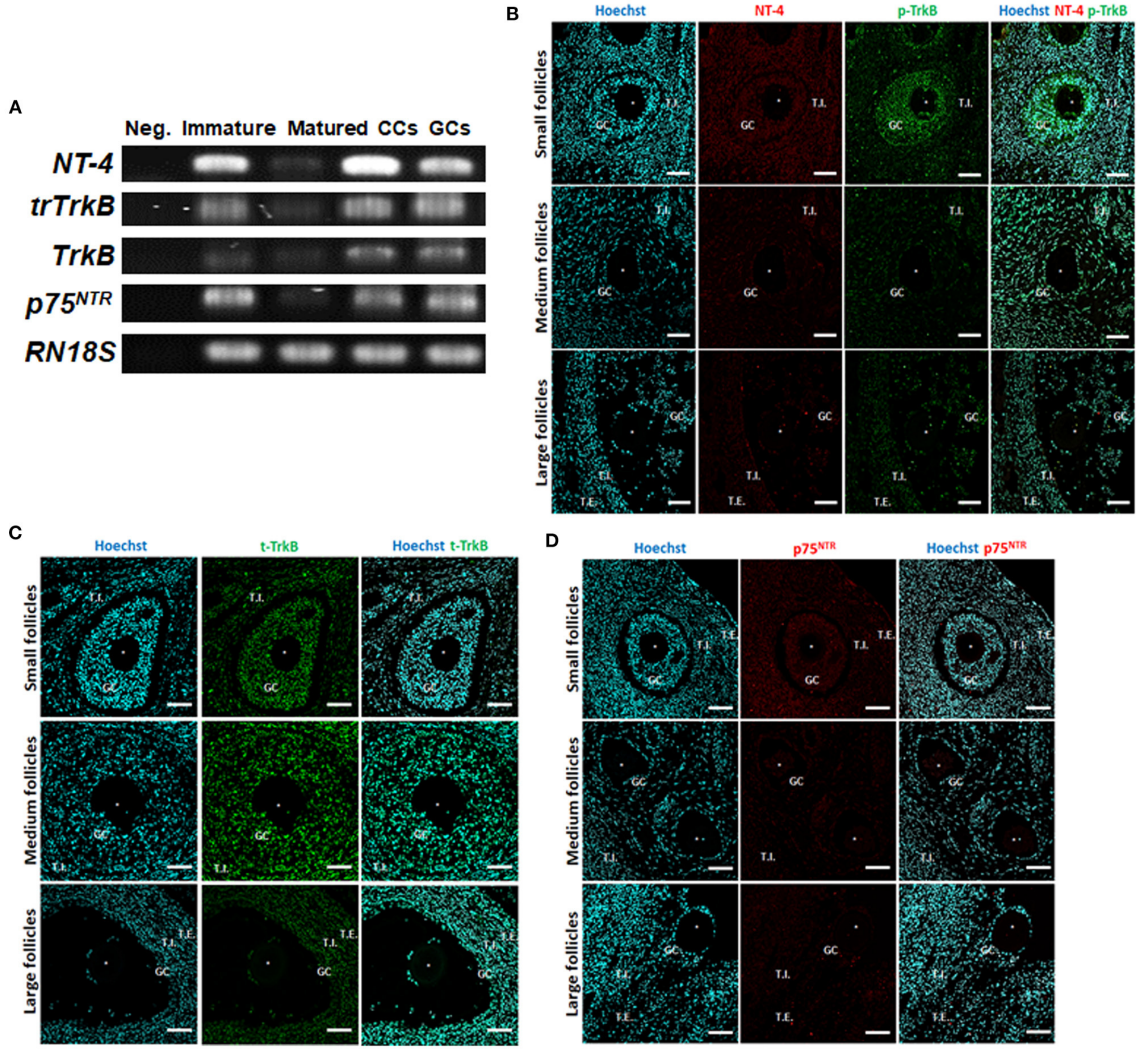
Identification and localization of neurotrophin-4 (NT-4) and its receptors in porcine ovarian follicular cells by RT-PCR and fluorescent immunohistochemistry. **(A)** Expression of *NT-4*, full-length *tropomyosin receptor kinase B* (*TrkB*), *truncated TrkB* (*trTrkB*), and *pan-neurotrophin receptor* (*p75*^*NTR*^) mRNAs in porcine ovarian follicular cells, immature and matured oocytes was determined via RT-PCR. *RN18S* served as a ubiquitously expressed control. Distilled water was used as the negative control (Neg.). CCs, Cumulus cells; GCs, Granulosa cells. This experiment was replicated at least three times. **(B–D)** Identification and localization of NT-4 and its receptors in porcine ovaries via fluorescent immunohistochemistry. Pig ovarian tissues were divided into small (1–2 mm), medium (3–6 mm), and large (7–9 mm) sections according to follicle size. **(B)** NT-4 and Phospho-TrkB (p-TrkB). **(C)** Total TrkB (t-TrkB). **(D)** p75^NTR^. T.I., Theca interna cells; T.E., Theca externa cells. Asterisks represent ooplasm.

We first examined the expression of NT-4 and its related receptor genes in porcine follicular cells using RT-PCR analysis. Distilled water was used as the negative control. The mRNA transcripts of *NT-4*, full-length *TrkB, truncated TrkB* (*trTrkB*), and *p75*^*NTR*^ were detected in all porcine follicular cells containing GCs, CCs, immature, and matured (MII) oocytes (Figure 1A).

To identify the location of NT-4 and its receptors in the porcine ovary according to follicle size (small, medium, and large), we conducted IHC analysis using paraffin-embedded ovarian sections. NT-4 was mainly expressed in theca interna cells, theca externa cells, and GCs, whereas phospho-TrkB (p-TrkB) was mainly expressed in GCs, CCs, and oocytes (Figure 1B). Total TrkB (t-TrkB) was strongly expressed in theca interna cells, theca externa cells, GCs, and CCs (Figure 1C). p75^NTR^ was expressed in all follicular cells (Figure 1D).

### Effects of NT-4 Supplementation During IVM on Nuclear Maturation of Oocytes and CC Expansion in Porcine COCs

Since both NT-4 and EGF can activate RTKs (44), we determined the effect of NT-4 addition with or without EGF during IVM. In the case of NT-4 treatment without EGF, there was no significant difference in the nuclear maturation rate and the extent of CC expansion between the NT-4 treatment groups and the control group (Supplementary Figure 1).

**Figure 2:**
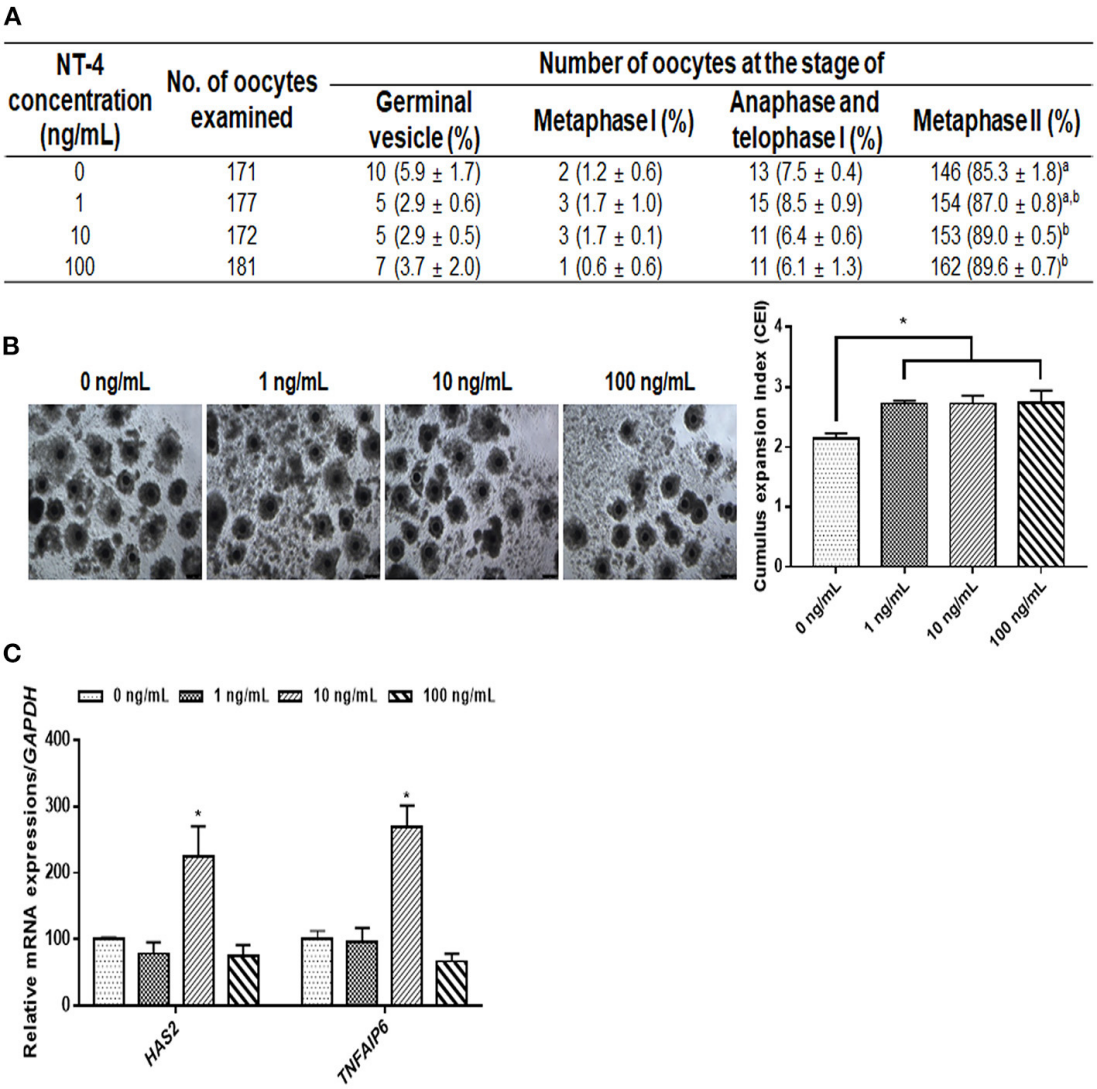
Effect of neurotrophin-4 (NT-4) treatment with epidermal growth factor (EGF) during *in vitro* maturation (IVM) of oocytes and cumulus cell (CC) expansion in porcine cumulus-oocytes complexes (COCs). **(A)** Evaluation of nuclear maturation rate in porcine COCs treated with EGF and NT-4. ^a,b^Values in the same column with different superscripts significantly differ (*p* < 0.05). **(B)** Morphological CC expansion of COCs treated with EGF and NT-4 during 42 h of IVM. The degree of CC expansion was examined using the cumulus scoring system: 0 (no expansion) to +4 (maximum expansion). Scale bars = 200 μm. **(C)** Expression of cumulus expansion-related genes (*HAS2* and *TNFAIP6*) in CCs treated with EGF and NT-4 during 42 h of IVM. The mRNA levels were normalized to *GAPDH* expression as a control. All data are expressed as the mean ± SEM. All experiments were replicated with three times. Asterisks indicate statistical significance (^*^p < 0.05).

During IVM, we investigated the effects of NT-4 on meiotic maturation and CC expansion. Different concentrations of NT-4 (0, 1, 10, and 100 ng/mL) were treated with EGF (10 ng/mL) to evaluate the nuclear maturation rate during IVM (Figure 2A). The nuclear maturation rate significantly increased in the 10 ng/mL (89.0 ± 0.5%) and 100 ng/mL (89.6 ± 0.7%) groups compared to the control group (85.3 ± 1.8%).

Morphological and gene expression analyses of CC expansion were performed to identify the effect of NT-4 supplementation in the presence of EGF in the IVM medium. The morphological changes due to CC expansion showed that the CC expansion index significantly increased in all groups treated with NT-4 compared to the control group (Figure 2B). The CC expansion-related gene expression analysis showed that *HAS2* and *TNFAIP6* transcript levels significantly increased in the 10 ng/mL NT-4-treated group (Figure 2C). Therefore, NT-4 supplementation in the presence of 10 ng/mL EGF during IVM can improve the nuclear maturation of porcine oocytes and CC expansion.

### Effects of NT-4 Supplementation During IVM on Cytoplasmic Maturation of Porcine Oocytes

**Figure 3:**
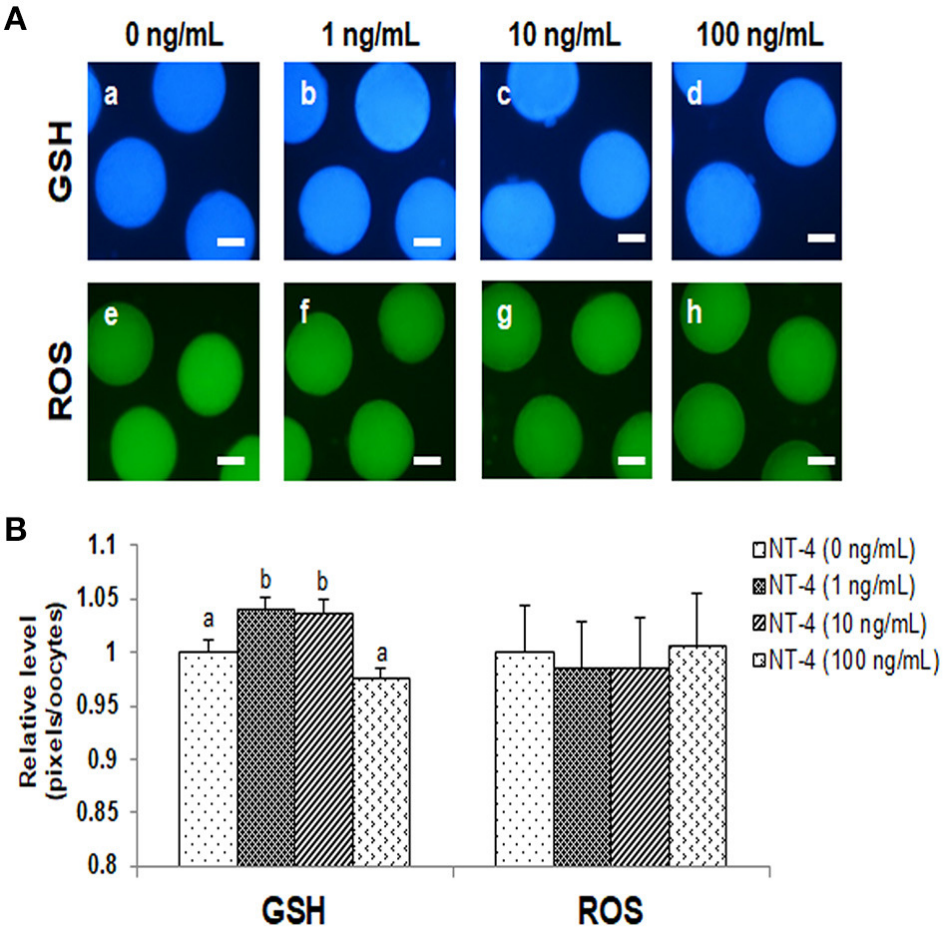
Epifluorescent photomicrographic images of *in vitro* matured porcine oocytes. **(A)** Oocytes were stained with CellTracker Blue (a–d) and 2′, 7′-dichlorodihydro-fluorescein diacetate (H_2_DCFDA) (e–h) to detect intracellular levels of glutathione (GSH) and reactive oxygen species (ROS), respectively. **(B)** Effect of NT-4 in maturation medium on intracellular GSH and ROS levels in *in vitro* matured porcine oocytes. Bars with different letters (a, b) represent significantly different values (*p* < 0.05). Number of GSH samples = 80, Number of ROS samples = 80. The experiment was performed four times. Scale bars = 50 μm.

The intracellular GSH levels in the 1 and 10 ng/mL NT-4-treated groups were significantly higher than those in the control group. However, there was no significant difference in the intracellular ROS levels among the groups. Thus, the NT-4 treatment during IVM did not affect intracellular ROS levels in mature oocytes, but the 1 and 10 ng/mL NT-4-treated groups had significantly improved intracellular GSH levels (Figure 3).

### Effects of NT-4 Supplementation During IVM on Specific Gene Expression in Matured Oocytes and CCs

**Figure 4:**
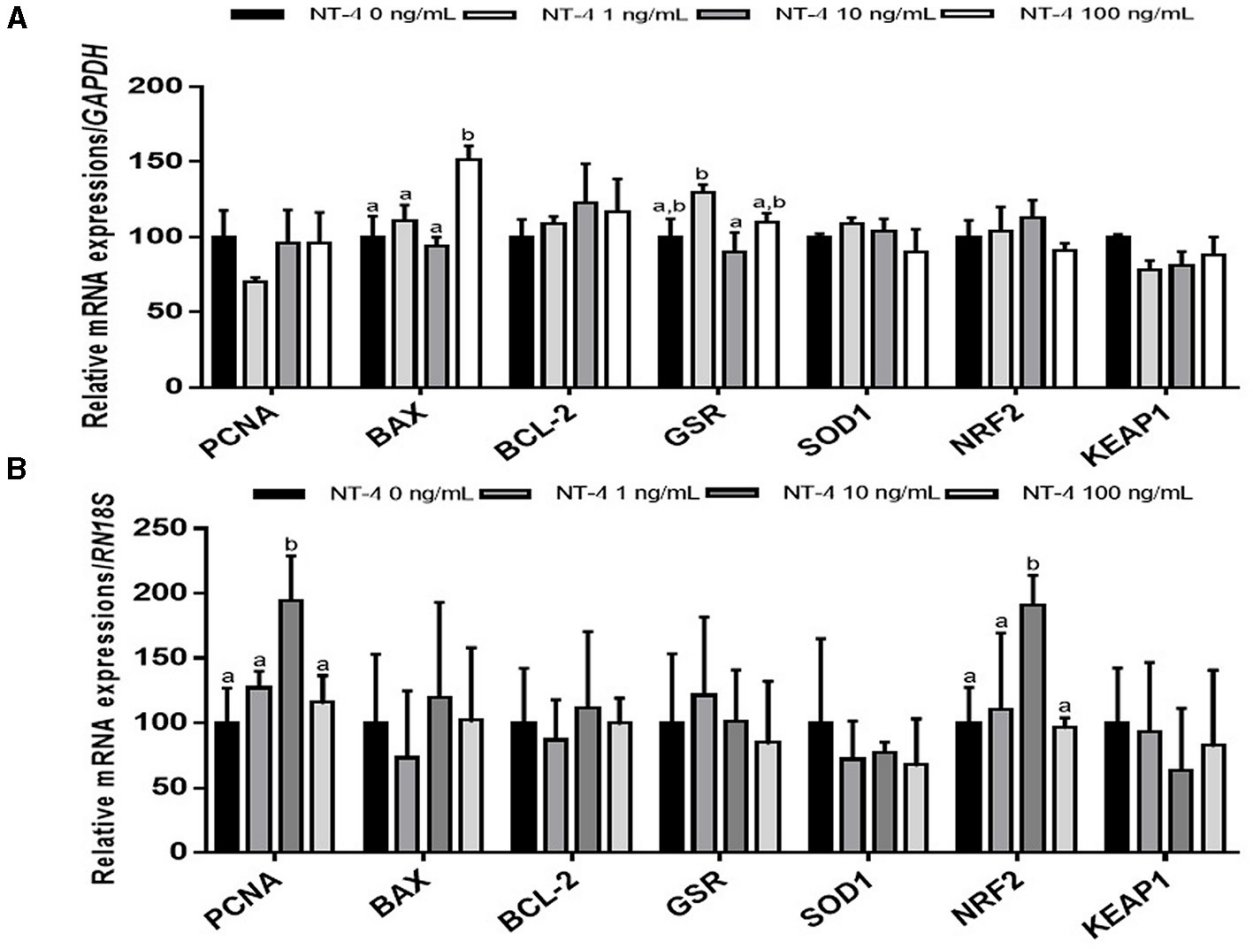
Expression levels of proliferation, apoptosis, and antioxidant-related genes in neurotrophin-4 (NT-4)-treated cumulus cells and oocytes. Mean ± SEM expression of *PCNA, BAX, BCL-2, GSR, SOD1, NRF2*, and *KEAP1* mRNA in **(A)** cumulus cells and **(B)** oocytes. This experiment was replicated three times. Within each end point, bars with different letters (a and b) are significantly (*p* < 0.05) different.

To identify why the IVM efficiency was improved when NT-4 was added to the IVM medium, matured oocytes and CCs were separated from COCs and used to examine the transcript levels compared to the control group. In matured CCs after IVM, the mRNA transcription level of the pro-apoptotic gene *BAX* was significantly higher in the 100 ng/mL NT-4-supplemented group than in the other groups. The transcription level of glutathione reductase gene *GSR* was not significantly different between the NT-4 treatment groups and the control, but the transcription level of *GSR* in the 10 ng/mL NT-4-supplemented group was significantly higher than that in the 1 ng/mL NT-4 group (Figure 4A).

In matured oocytes, the expression levels of the cell proliferation-related gene *PCNA* and antioxidant response-related gene *NRF2* significantly increased in the 10 ng/mL NT-4-supplemented group (Figure 4B).

**Figure 5:**
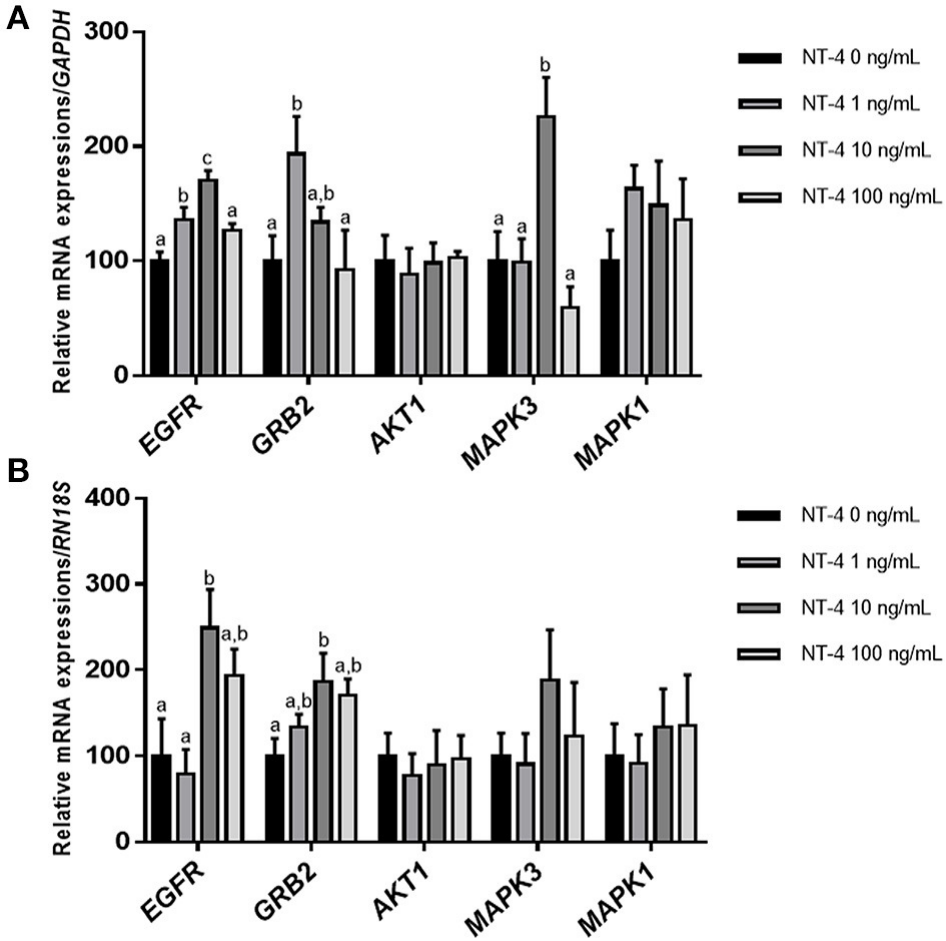
Expression levels of epidermal growth factor receptor (EGFR) signaling pathway-related genes in neurotrophin-4 (NT-4)-treated cumulus cells and oocytes. Mean ± SEM expression of *EGFR, GRB2, AKT1, MAPK3* (*ERK1*), and *MAPK1* (*ERK2*) mRNA in **(A)** cumulus cells and **(B)** oocytes. This experiment was replicated at least three times. Within each end point, bars with different letters (a, b, and c) are significantly (*p* < 0.05) different.

To determine whether the addition of NT-4 to the IVM medium affects the expression level of EGFR-related genes, the mRNA transcript levels of *EGFR, GRB2, AKT1, MAPK3* (*ERK1*), and *MAPK1* (*ERK2*) were investigated in matured CCs and oocytes from each group. In matured CCs, the transcription levels of *EGFR* and *MAPK3* were significantly higher in the group treated with 10 ng/mL NT-4 than in the control group. The 1 ng/mL NT-4-treated group showed significantly higher mRNA expression levels of *EGFR* and *GRB2* (growth factor receptor bound protein 2) than the control group (Figure 5A). In contrast, in matured oocytes, only the 10 ng/mL NT-4 treatment group showed significantly higher mRNA transcript levels of *EGFR* and *GRB2* than the control group (Figure 5B).

### Effects of NT-4 Supplementation During IVM on Embryonic Developmental Competence After PA

**Figure 6:**
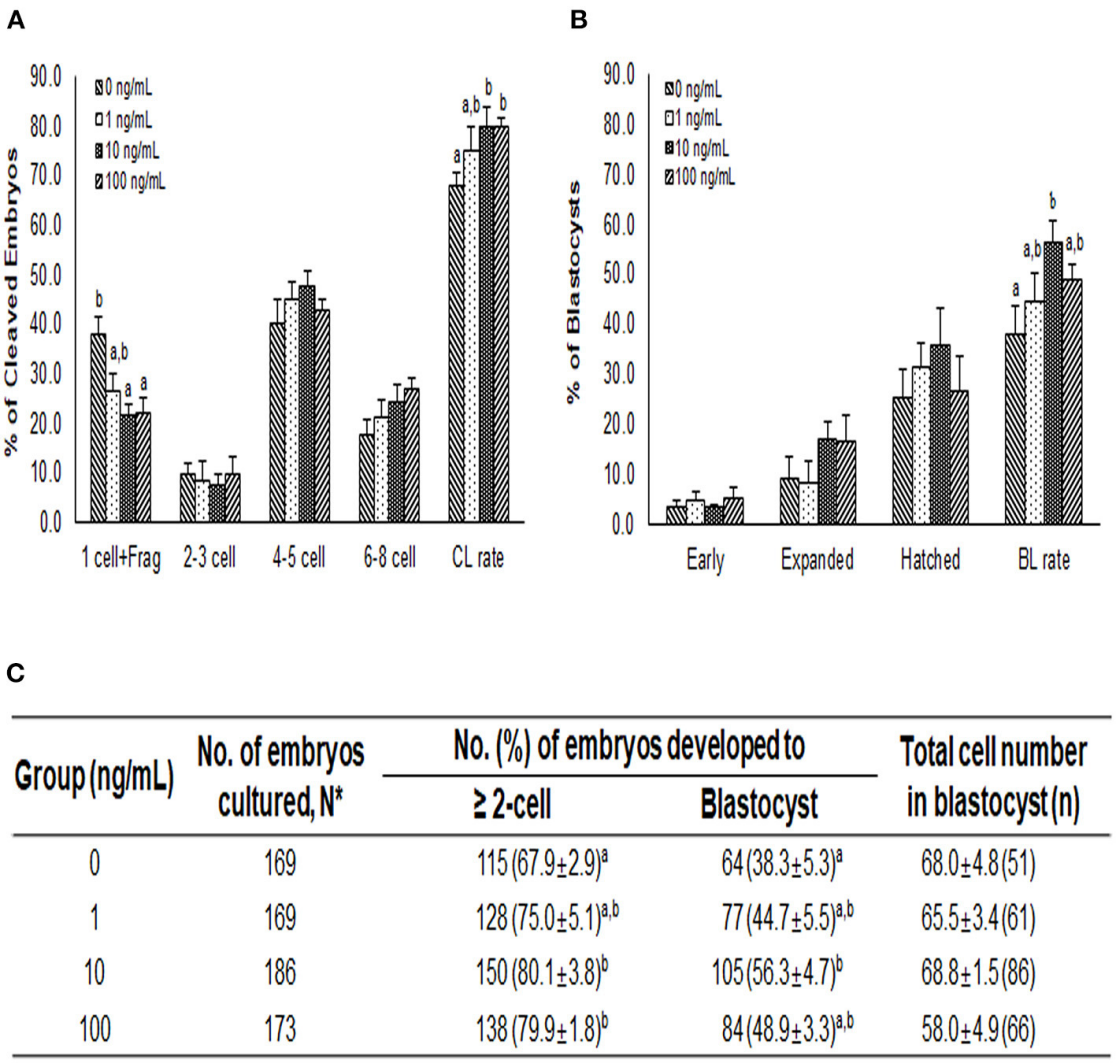
Effect of NT-4 treatment during *in vitro* maturation (IVM) on embryonic development after parthenogenetic activation (PA) in terms of **(A)** the cleavage pattern and **(B)** the blastocyst formation pattern of the PA embryos. Within each end point, bars with different letters (a, b) are significantly (*p* < 0.05) different for different concentrations of NT-4 treatment. CL, cleavage; BL, blastocyst. **(C)** Summary of embryonic development after PA. The cleavage rate was measured on day 2, and the blastocyst formation rate was evaluated on day 7 of culture. *N: Four times replicated.

After PA, the cleavage rates were significantly higher in the 10 and 100 ng/mL NT-4 groups than in the control group. The cleavage pattern on day 2 showed that the ratio of one cell and fragmentation group (1 cell + Frag) was significantly lower in the 10 and 100 ng/mL NT-4 groups than in the control group (Figure 6A). On day 7, blastocyst formation rates were significantly higher in the 10 ng/mL NT-4 group (Figure 6B). However, there was no significant difference in the total number of blastocysts between the control and NT-4 treatment groups (Figure 6C).

## Discussion

The neurotrophin family promotes growth, survival, and development of neurons (45). Moreover, it plays an important role in the development of the ovaries, where various nerves are distributed. Especially, NT-4 is known to accelerate the follicular assembly in rodents and humans (18, 29–31). During ovarian development in mammals, sympathetic and sensory nerve fibers can reach most of the ovarian tissues, including follicles and interstitial tissues, to facilitate ovarian development (46). Therefore, various neurotrophic factors that exist in intra-ovarian follicular cells can promote the growth of theca cells (TCs), GCs, CCs, and oocytes (19, 47). Many studies have shown that BDNF and GDNF, which belong to the neurotrophin family, are involved in oocyte maturation (27, 36, 37, 48, 49). However, it is not yet known whether NT-4 is related to oocyte maturation and follicular development in pigs. Therefore, we investigated the effect of NT-4 supplementation on porcine COCs during IVM.

Numerous studies have shown that the BDNF-TrkB signaling pathway is important for oogenesis (31), follicular development (50), oocyte maturation (51, 52), embryonic development (53, 54) and placental development (55). However, there are few reports on the relationship between the NT-4-TrkB signaling pathway in the mammalian female reproductive system. One study reported that NT-4 can enhance *in vitro* follicular assembly during human fetal ovarian development (32). Thus, to determine whether NT-4 acts during porcine follicular development, we first identified the localization and expression of NT-4 and its receptors (TrkB and p75^NTR^) in porcine ovarian follicular cells. As a result of immunofluorescence staining of the paraffin-embedded ovarian tissue, NT-4 was mainly expressed in TCs and GCs, whereas p-TrkB was mainly expressed in GCs, CCs, and oocytes. T-TrkB was strongly expressed in TCs, GCs, and CCs; p75^NTR^ was expressed in all follicular cells. At the mRNA transcript levels, *NT-4*, full-length *TrkB, trTrkB*, and *p75*^*NTR*^ were expressed in all porcine follicular cells containing GCs, CCs, and immature and matured oocytes. Previous study has reported the identification of NGF and its receptors (Tropomyosin receptor kinase A; TRKA and p75^NTR^) in the porcine ovary (56). They suggested NGF and TRKA proteins were most highly expressed in TCs and GCs of large follicles among the antral follicles. On the other hand, p75^NTR^ protein was strongly expressed in all antral follicles regardless of follicle size. In the present study, although p-TrkB and p75^NTR^ appear to be expressed more strongly in small follicle-derived cells, but they could not be quantified. Instead, it was confirmed that they were present in all antral follicle-derived cells regardless of follicle size. Therefore, our findings indicate that NT-4 and its receptors are required for porcine follicular development. Another studies have demonstrated via enzyme-linked immunosorbent assays that NT-4 is present in human follicular fluid (397.0 ± 71 pg/mL) (21) and reported the presence of NT-4 and TrkB in human CCs (23). Similar to those studies, we also identified that NT-4 and its receptors are generally expressed in porcine oocytes and ovarian follicular cells. These results suggest that NT-4 plays an essential role in the development of ovarian follicular cells in pigs.

In the present study, we demonstrated that the NT-4 supplementation during IVM is beneficial for oocyte maturation and subsequent embryonic development after PA. Since NT-4 and EGF can activate RTKs such as TrkB and EGFR, respectively, the nuclear maturation rate was evaluated by classifying the EGF-treated groups and non-treated groups in which NT-4 was present in the IVM medium. When EGF was absent in the IVM medium, there was no significant difference in the nuclear maturation rate in any group, regardless of NT-4 treatment. However, when EGF was present in the IVM medium and NT-4 was used at various concentrations, the nuclear maturation rate significantly increased in the 10 and 100 ng/mL NT-4 treatment groups compared to the control. In a mouse study, NT-4 was shown to improve oocyte nuclear maturation; the first polar body extrusion rate was significantly higher in the NT-4 treatment groups at 1 and 10 ng/mL than in the control group (21). Lee et al. reported that simultaneous treatment with EGF (10 ng/mL) and BDNF (30 ng/mL) in the porcine IVM medium did not significantly contribute to promoting the meiotic progression of porcine oocytes, but it did enhance the capacity of subsequent embryonic development after *in vitro* fertilization and somatic cell nuclear transfer (25). Interestingly, in the present study, the addition of both NT-4 (10 ng/mL) and EGF (10 ng/mL) to the IVM medium improved the nuclear and cytoplasmic maturation of porcine oocytes, as well as their subsequent developmental competence after PA. Moreover, these findings suggest that NT-4 and EGF improve porcine oocytes maturation *in vitro* by binding to RTKs synergistically rather than competitively.

In this study, we performed nuclear maturation, morphological analysis of CC expansion, and cytoplasmic maturation after NT-4 treatment during IVM to understand the physiological role of NT-4 in pig ovaries. Generally, as the extracellular matrix structure is synthesized in CCs, CCs expand, and this process induces the successful maturation of the oocyte before ovulation (57). Moreover, intracellular GSH synthesis is crucial for the cytoplasmic maturation of oocytes because it protects cells from oxidative stress (58). During 42 h of IVM, the nuclear maturation rate significantly increased in the 10 and 100 ng/mL NT-4 treatment groups. In addition, the degree of CC expansion significantly increased in all NT-4 treatment groups, and the mRNA expression levels of hyaluronan synthase 2 (*HAS2*) and tumor necrosis factor alpha-induced protein 6 (*TNFAIP6*), which are the specific genes related to CC expansion (the indicator of synthesis of the extracellular matrix structure in CCs), significantly increased only in the 10 ng/mL NT-4 treatment group. The intracellular GSH levels in the cytoplasm of matured oocytes significantly increased in the 1 and 10 ng/mL NT-4 treatment groups. These findings suggest that the physiological role of NT-4 in pig ovaries is to promote oocyte maturation.

We also investigated whether NT-4 supplementation during IVM affects the expression of genes involved in cell proliferation (proliferating cell nuclear antigen, *PCNA*), apoptosis (B-cell lymphoma 2; *BCL-2*, and BCL2 Associated X; *BAX*), and antioxidative pathways (glutathione disulfide reductase; *GSR*, Superoxide dismutase1; *SOD1*, nuclear factor-erythroid factor 2-related factor 2; *NRF2*, and Kelch-like ECH associated protein 1; *KEAP1*) in matured oocytes and CCs. There was no significant difference in the expression levels of other transcripts (*PCNA, BCL-2, GSR, SOD1, NRF2*, and *KEAP1*) between matured CCs and the control, but only the *BAX* gene showed a significant increase in expression levels in the 100 ng/mL NT-4 treatment group. Mani et al. (59) showed that when insulin growth factor-1 (IGF-1), one of growth factors, was treated at 30 ng/mL or higher, the mRNA expression level of *BAX* increased in bovine granulosa cells. They suggested that a high dose of IGF-1 (>30 ng/mL) treatment induces apoptosis in bovine GCs (59). We also observed that NT-4 supplementation at a high concentration (100 ng/mL) during IVM increased the level of pro-apoptotic *BAX* mRNA expression. Therefore, it seems that treatment with a high concentration of NT-4 (>100 ng/mL) can induce apoptosis in porcine CCs. However, in contrast to the results in matured CCs, there was no significant difference in the mRNA expression level of *BAX* in matured oocytes; the mRNA expression levels of *PCNA* and *NRF2* increased only in the 10 ng/mL NT-4 treatment group. Therefore, the 10 ng/mL of NT-4 promotes the maturation of oocytes by increasing the transcription levels of *PCNA* and *NRF2* genes in oocytes.

Many studies have reported that the activation of the EGFR signaling pathway enhances the maturation of oocytes and the development of COCs (34, 60–62). Furthermore, some studies have reported that EGFR-ERK1/2 signaling activation is important for oocyte maturation (34), cumulus expansion (63), ovulation (64), luteinization (65), and fertilization (66). Based on the findings of these previous studies, we investigated whether treatment with NT-4 during IVM regulates the mRNA expression levels of other factors related to the EGFR signaling pathway. Our results showed that the mRNA transcription level of *EGFR* in matured CCs significantly increased in the 10 and 100 ng/mL NT-4 groups. The mRNA transcript level of *GRB2*, an adapter protein that binds to membrane receptors such as EGFR, increased in the 1 ng/mL NT-4 group. In contrast, the mRNA expression levels of *EGFR* and *GRB2* increased only in the 10 ng/mL NT-4 treatment group in matured oocytes. Therefore, we concluded that 10 ng/mL of NT-4 promotes porcine oocyte *in vitro* maturation by increasing the mRNA expression of *EGFR* in porcine COCs and interacting with EGFR-related signaling pathways.

The activation of *EGFR* in cancer cells is known to induce the activation of extracellular-regulated kinase (ERK) or Akt (protein kinase B; PKB) signaling pathways (67, 68). Thus, we investigated the mRNA expression levels of *ERK1/2* (also known as MAPK3/1) and *AKT1* in porcine COCs after IVM. There was no significant difference in the mRNA expression levels of *AKT1* and *ERK1/2* transcripts in any group of matured oocytes. However, in matured CCs, the transcription level of *MAPK3* (*ERK1*) was significantly higher only in the 10 ng/mL NT-4 group. Therefore, further studies are needed to confirm whether NT-4 supplementation of during IVM activates the EGFR-ERK signaling pathway in porcine COCs.

Taken together, the results of this study suggest that the supplementation of NT-4 during porcine IVM can promote oocyte maturation by interacting with the EGFR signaling pathway, and the optimal concentration of NT-4 in porcine IVM was determined to be 10 ng/mL. Moreover, we demonstrated for the first time that NT-4 can be considered a beneficial factor in porcine follicular development, oocyte maturation, and subsequent embryonic development after PA.

## Conclusions

This study demonstrates the identification and localization of NT-4, TrkB, and p75^NTR^ in porcine ovaries. Our findings showed that NT-4 and its receptors are involved in porcine follicular development and that NT-4 supplementation improves the nuclear and cytoplasmic maturation of porcine oocytes *in vitro* by interacting with the EGFR signaling pathway. Furthermore, NT-4 supplementation during IVM was shown to enhance the developmental potential of PA-derived porcine embryos. Therefore, NT-4 is required for porcine follicular development and oocyte maturation.

## Data Availability Statement

The original contributions presented in the study are included in the article/Supplementary Materials, further inquiries can be directed to the corresponding author/s.

## Ethics Statement

Ethical review and approval was not required for the animal study because it is not subject to review by the Animal Ethics Committee, as only the pig ovaries were taken from the slaughterhouse.

## Author Contributions

MK and S-HH: conceptualization, validation, writing-original draft preparation, and writing-review and editing. MK, S-UH, JY, JL, and EK: methodology. MK, LC, GK, HC, and DO: investigation. MK, S-UH, and JL: formal analysis. S-HH: funding acquisition. All authors have read and agreed to the published version of the manuscript.

## Funding

This work was supported, in part, by a grant from the National Research Foundation of Korea Grant funded by the Korean Government (2020R1A2C2008276), Korea Institute of Planning and Evaluation for Technology in Food, Agriculture, Forestry and Fisheries (IPET) through Agri-Bio industry Technology Development Program (grant number: 318016-5) and Agriculture, Food and Rural Affairs Convergence Technologies Program for Educating Creative Global Leader (grant number: 320005-4), funded by Ministry of Agriculture, Food and Rural Affairs (MAFRA) and The Global Research and Development Center (GRDC) Program through the National Research Foundation of Korea (NRF) funded by the Ministry of Education, Science and Technology (2017K1A4A3014959), Republic of Korea.

## Conflict of Interest

The authors declare that the research was conducted in the absence of any commercial or financial relationships that could be construed as a potential conflict of interest.

## Publisher's Note

All claims expressed in this article are solely those of the authors and do not necessarily represent those of their affiliated organizations, or those of the publisher, the editors and the reviewers. Any product that may be evaluated in this article, or claim that may be made by its manufacturer, is not guaranteed or endorsed by the publisher.

## Supplementary Material

Supplementary Figure 1 Effect of neurotrophin-4 (NT-4) treatment without epidermal growth factor (EGF) during *in vitro* maturation (IVM) of oocytes and cumulus cell (CC) expansion in porcine cumulus-oocytes complexes (COCs). **(A)** Evaluation of nuclear maturation rate in porcine COCs treated with NT-4 in the absence of EGF during IVM. ^a,b^Values in the same column with different superscripts significantly differ (*p* < 0.05). **(B)** Morphological CC expansion of COCs treated with NT-4 in the absence of EGF during 42 h of IVM. The degree of CC expansion was examined using the cumulus scoring system: 0 (no expansion) to +4 (maximum expansion). Scale bars = 200 μm. **(C)** Expression of cumulus expansion-related genes (*HAS2* and *TNFAIP6*) in CCs treated with NT-4 in the absence of EGF during 42 h of IVM. The mRNA levels were normalized to *GAPDH* expression as a control. All data are expressed as the means ± SEM. All experiments were replicated three times.

Supplementary Table 1 Primer lists for RT-PCR.

Supplementary Table 2 Antibody lists for fluorescent immunohistochemistry.

Supplementary Table 3 Primer lists for qRT-PCR.

## References

[1] Paterson L DeSousa P Ritchie W King T Wilmut I. Application of reproductive biotechnology in animals: implications and potentials: applications of reproductive cloning. Anim Reprod Sci. (2003) 79:137–43. 10.1016/S0378-4320(03)00161-1 14643101

[2] Seidel G Jr. Reproductive biotechnology and “big” biological questions. Theriogenology. (2000) 53:187–94. 10.1016/S0093-691X(99)00251-4 10735073

[3] Niemann H Rath D. Progress in reproductive biotechnology in swine. Theriogenology. (2001) 56:1291–304. 10.1016/S0093-691X(01)00630-6 11758883

[4] Long J. Reproductive biotechnology and gene mapping: tools for conserving rare breeds of livestock. Reprod Domest Anim. (2008) 43:83–8. 10.1111/j.1439-0531.2008.01146.x 18638108

[5] Hwang S-U Jeon Y Yoon JD Cai L Kim E Yoo H. Effect of ganglioside GT1b on the *in vitro* maturation of porcine oocytes and embryonic development. J Reprod Dev. (2015) 61:549–57. 10.1262/jrd.2015-049 26370787 PMC4685221

[6] Yoon JD Hwang S-U Kim E Jin M Kim S Hyun S-H. GDF8 activates p38 MAPK signaling during porcine oocyte maturation *in vitro*. Theriogenology. (2017) 101:123–34. 10.1016/j.theriogenology.2017.06.003 28708509

[7] Kim E Jeon Y Kim DY Lee E Hyun S-H. Antioxidative effect of carboxyethylgermanium sesquioxide (Ge-132) on IVM of porcine oocytes and subsequent embryonic development after parthenogenetic activation and IVF. Theriogenology. (2015) 84:226–36. 10.1016/j.theriogenology.2015.03.006 25913277

[8] Kwak S-S Cheong S-A Jeon Y Lee E Choi K-C Jeung E-B. The effects of resveratrol on porcine oocyte *in vitro* maturation and subsequent embryonic development after parthenogenetic activation and *in vitro* fertilization. Theriogenology. (2012) 78:86–101. 10.1016/j.theriogenology.2012.01.024 22445189

[9] Yoon JD Hwang S-U Kim M Jeon Y Hyun S-H. Growth differentiation factor 8 regulates SMAD2/3 signaling and improves oocyte quality during porcine oocyte maturation *in vitro*. Biol Reprod. (2019) 101:63–75. 10.1093/biolre/ioz066 31004472

[10] Abeydeera L. *In vitro* production of embryos in swine. Theriogenology. (2002) 57:257–273. 10.1016/S0093-691X(01)00670-7 11775974

[11] Field SL Dasgupta T Cummings M Orsi MN. Cytokines in ovarian folliculogenesis, oocyte maturation and luteinisation. Mol Reprod Dev. (2014) 81:284–314. 10.1002/mrd.22285 24273059

[12] Pawlak P Warzych E Cieslak A Malyszka N Maciejewska E Madeja ZE. The consequences of porcine IVM medium supplementation with follicular fluid become reflected in embryo quality, yield and gene expression patterns. Sci Rep. (2018) 8:1–12. 10.1038/s41598-018-33550-4 30333518 PMC6193000

[13] Lee Y-M Kumar BM Lee J-H Lee W-J Kim T-H Lee S-L. Characterisation and differentiation of porcine ovarian theca-derived multipotent stem cells. Vet J. (2013) 197:761–8. 10.1016/j.tvjl.2013.04.011 23702282

[14] Yoon JD Jeon Y Cai L Hwang S-U Kim E Lee E. Effects of coculture with cumulus-derived somatic cells on *in vitro* maturation of porcine oocytes. Theriogenology. (2015) 83:294–305. 10.1016/j.theriogenology.2014.09.025 25442018

[15] Henderson CE. Role of neurotrophic factors in neuronal development. Curr Opin Neurobiol. (1996) 6:64–70. 10.1016/S0959-4388(96)80010-9 8794045

[16] Ernfors P. Local and target-derived actions of neurotrophins during peripheral nervous system development. Cell Mol Life Sci. (2001) 58:1036–44. 10.1007/PL00000918 11529496 PMC11337391

[17] Dissen G Hirshfield AN Malamed S Ojeda S. Expression of neurotrophins and their receptors in the mammalian ovary is developmentally regulated: changes at the time of folliculogenesis. Endocrinology. (1995) 136:4681–92. 10.1210/endo.136.10.7664689 7664689

[18] Dissen GA Romero C Paredes A Ojeda RS. Neurotrophic control of ovarian development. Microsc Res Techn. (2002) 59:509–15. 10.1002/jemt.10227 12467027

[19] Dissen GA Garcia-Rudaz C Ojeda RS. Role of neurotrophic factors in early ovarian development. Semin Reprod Med. (2009) 27:24–1. 10.1055/s-0028-1108007 19197802 PMC3525518

[20] Skaper SD. The neurotrophin family of neurotrophic factors: an overview. Neurotrophic Factors. (2012) 846:1–12. 10.1007/978-1-61779-536-7_1 22367796

[21] Seifer DB Feng B Shelden RM Chen S Dreyfus FC. Neurotrophin-4/5 and neurotrophin-3 are present within the human ovarian follicle but appear to have different paracrine/autocrine functions. J Clin Endocrinol Metab. (2002) 87:4569–71. 10.1210/jc.2002-020499 12364436

[22] Ibanez C Hallbook F Godeau F Persson H. Expression of neurotrophin-4 mRNA during oogenesis in *Xenopus laevis*. nt J Dev Biol. (2002) 36:239–45. 1525011

[23] Seifer DB Feng B Shelden MR. Immunocytochemical evidence for the presence and location of the neurotrophin–Trk receptor family in adult human preovulatory ovarian follicles. Am J Obstetr Gynecol. (2006) 194:1129–34. 10.1016/j.ajog.2005.12.022 16580310

[24] Barbacid M. Neurotrophic factors and their receptors. Curr Opin Cell Biol. (1995) 7: 48–155. 10.1016/0955-0674(95)80022-0 7612265

[25] Lee E Jeong YI Park SM Lee JY Kim JH Park SW. Beneficial effects of brain-derived neurotropic factor on *in vitro* maturation of porcine oocytes. Reproduction. (2007) 134:405–14. 10.1530/REP-06-0288 17709559

[26] Anderson RA Bayne RA Gardner J De Sousa AP. Brain-derived neurotrophic factor is a regulator of human oocyte maturation and early embryo development. Fertil Steril. (2010) 93:1394–406. 10.1016/j.fertnstert.2009.04.007 19463996

[27] Zhao X Du F Liu X Ruan Q Wu Z Lei C. Brain-derived neurotrophic factor (BDNF) is expressed in buffalo (*Bubalus bubalis*) ovarian follicles and promotes oocyte maturation and early embryonic development. Theriogenology. (2019) 130:79–88. 10.1016/j.theriogenology.2019.02.020 30877846

[28] Pepling ME. Follicular assembly: mechanisms of action. Reproduction. (2012) 143:139. 10.1530/REP-11-0299 22065859

[29] Ojeda SR Romero C Tapia V Dissen AG. Neurotrophic and cell–cell dependent control of early follicular development. Mol Cell Endocrinol. (2000) 163:67–71. 10.1016/S0303-7207(99)00242-7 10963876

[30] Harel S Jin S Fisch B Feldberg D Krissi H Felz C. Tyrosine kinase B receptor and its activated neurotrophins in ovaries from human fetuses and adults. Mol Hum Reprod. (2006) 12:357–65. 10.1093/molehr/gal033 16648150

[31] Anderson RA Robinson LL Brooks J Spears N. Neurotropins and their receptors are expressed in the human fetal ovary. J Clin Endocrinol Metab. (2002) 87:890–7. 10.1210/jcem.87.2.8221 11836338

[32] Farhi J Fisch B Garor R Peled Y Pinkas H Abir R. Neurotrophin 4 enhances in vitro follicular assembly in human fetal ovaries. Fertil Steril. (2011) 95:1267–71. 10.1016/j.fertnstert.2010.03.051 20447632

[33] De Luca A Carotenuto A Rachiglio A Gallo M Maiello MR Aldinucci D. The role of the EGFR signaling in tumor microenvironment. J Cell Physiol. (2008) 214:559–67. 10.1002/jcp.21260 17894407

[34] Jamnongjit M Gill A Hammes RS. Epidermal growth factor receptor signaling is required for normal ovarian steroidogenesis and oocyte maturation. Proc Natl Acad Sci. (2005) 102:16257–62. 10.1073/pnas.0508521102 16260720 PMC1283479

[35] Vigneswara V Kundi S Ahmed Z. Receptor tyrosine kinases: molecular switches regulating CNS axon regeneration. J Signal Transd. (2012) 2012: 361721. 10.1155/2012/361721 22848811 PMC3405719

[36] Linher K Wu D Li J. Glial cell line-derived neurotrophic factor: an intraovarian factor that enhances oocyte developmental competence *in vitro*. Endocrinology. (2007) 148:4292–301. 10.1210/en.2007-0021 17540724

[37] Valleh MV Rasmussen MA Hyttel P. Combination effects of epidermal growth factor and glial cell line-derived neurotrophic factor on the *in vitro* developmental potential of porcine oocytes. Zygote. (2016) 24:465. 10.1017/S0967199415000416 26350562

[38] Hwang S-U Yoon JD Kim M Cai L Choi H Oh D. R-Spondin 2 and WNT/CTNNB1 signaling pathways are required for porcine follicle development and *in vitro* maturation. Animals. (2021) 11:709. 10.3390/ani11030709 33807916 PMC7998564

[39] Vanderhyden BC Caron PJ Buccione R Eppig JJ. Developmental pattern of the secretion of cumulus expansion-enabling factor by mouse oocytes and the role of oocytes in promoting granulosa cell differentiation. Dev Biol. (1990) 140:307–17. 10.1016/0012-1606(90)90081-S 2115479

[40] You J Kim J Lim J Lee E. Anthocyanin stimulates *in vitro* development of cloned pig embryos by increasing the intracellular glutathione level and inhibiting reactive oxygen species. Theriogenology. (2010) 74:777–85. 10.1016/j.theriogenology.2010.04.002 20537699

[41] Livak KJ Schmittgen DT. Analysis of relative gene expression data using real-time quantitative PCR and the 2– ΔΔCT method. Methods. (2001) 25:402–408. 10.1006/meth.2001.1262 11846609

[42] Lee J Park J-I Im Yun J Lee Y Yong H Lee ST. Rapamycin treatment during *in vitro* maturation of oocytes improves embryonic development after parthenogenesis and somatic cell nuclear transfer in pigs. J Vet Sci. (2015) 16:373–80. 10.4142/jvs.2015.16.3.373 25797293 PMC4588024

[43] Yoshioka K Suzuki C Tanaka A Anas M-K I Iwamura S. Birth of piglets derived from porcine zygotes cultured in a chemically defined medium. Biol Reprod. (2002) 66:112–9. 10.1095/biolreprod66.1.112 11751272

[44] Glass DJ Yancopoulos DG. The neurotrophins and their receptors. Trends Cell Biol. (1993) 3:262–8. 10.1016/0962-8924(93)90054-5 14731744

[45] Gillespie LN. Regulation of axonal growth and guidance by the neurotrophin family of neurotrophic factors. Clin Exp Pharmacol Physiol. (2003) 30:724–33. 10.1046/j.1440-1681.2003.03909.x 14516410

[46] Lara H McDonald J Ahmed C Ojeda S. Guanethidine-mediated destruction of ovarian sympathetic nerves disrupts ovarian development and function in rats. Endocrinology. (1990) 127:2199–209. 10.1210/endo-127-5-2199 1977580

[47] Ojeda SR Dissen GA Malamed S Hirshfield NA. A role for neurotrophic factors in ovarian development. In: Hsueh AJW Schomberg DW editors. Ovarian Cell Interactions. Proceedings in the Serono Symposia, USA Series. New York, NY: Springer (1993). 10.1007/978-1-4613-8336-9_14

[48] Cui L Fang L Mao X Chang H-M Leung PC Ye Y. GDNF-induced downregulation of miR-145-5p enhances human oocyte maturation and cumulus cell viability. J Clin Endocrinol Metab. (2018) 103:2510–21. 10.1210/jc.2017-02742 29897461

[49] Wang D-H Zhou H-X Liu S-J Zhou C-J Kong X-W Han Z. Glial cell line-derived neurotrophic factor supplementation promotes bovine *in vitro* oocyte maturation and early embryo development. Theriogenology. (2018) 113:92–101. 10.1016/j.theriogenology.2018.02.015 29477014

[50] Linher-Melville K Li J. The roles of glial cell line-derived neurotrophic factor, brain-derived neurotrophic factor and nerve growth factor during the final stage of folliculogenesis: a focus on oocyte maturation. Reproduction. (2013) 145:R43–54. 10.1530/REP-12-0219 23166367

[51] Kawamura K Kawamura N Mulders SM Gelpke MDS Hsueh JA. Ovarian brain-derived neurotrophic factor (BDNF) promotes the development of oocytes into preimplantation embryos. Proc Natl Acad Sci. (2005) 102:9206–11. 10.1073/pnas.0502442102 15967989 PMC1166611

[52] Zhao P Qiao J Huang S Zhang Y Liu S Yan L-Y. Gonadotrophin-induced paracrine regulation of human oocyte maturation by BDNF and GDNF secreted by granulosa cells. Hum Reprod. (2011) 26:695–702. 10.1093/humrep/deq390 21227937

[53] Kawamura K Kawamura N Fukuda J Kumagai J Hsueh AJ Tanaka T. Regulation of preimplantation embryo development by brain-derived neurotrophic factor. Dev Biol. (2007) 311:147–58. 10.1016/j.ydbio.2007.08.026 17880937

[54] Yi K Zhou X Shi D Chen H Qin Q Chen Y. The mRNA expression of brain-derived neurotrophic factor in oocytes and embryos and its effects on the development of early embryos in cattle. Animal. (2008) 2:1786–94. 10.1017/S1751731108002838 22444085

[55] Garcés MF Sanchez E Torres-Sierra AL Ruíz-Parra AI Angel-Müller E Alzate JP. Brain-derived neurotrophic factor is expressed in rat and human placenta and its serum levels are similarly regulated throughout pregnancy in both species. Clin Endocrinol. (2014) 81:141–51. 10.1111/cen.12391 24372023

[56] Jana B Koszykowska M Czarzasta J. Expression of nerve growth factor and its receptors, TrkA and p75, in porcine ovaries. J Reprod Dev. (2011) 57:468–74. 10.1262/jrd.10-180H 21502727

[57] Russell DL Robker LR. Molecular mechanisms of ovulation: co-ordination through the cumulus complex. Hum Reprod Update. (2007) 13:289–312. 10.1093/humupd/dml062 17242016

[58] Luberda Z. The role of glutathione in mammalian gametes. Reprod Biol. (2005) 5:5–17. 15821775

[59] Mani AM Fenwick MA Cheng Z Sharma MK Singh D Wathes CD. IGF1 induces up-regulation of steroidogenic and apoptotic regulatory genes via activation of phosphatidylinositol-dependent kinase/AKT in bovine granulosa cells. Reproduction. (2010) 139:139. 10.1530/REP-09-0050 19819918

[60] Nagyova E Camaioni A Scsukova S Mlynarcikova A Prochazka R Nemcova L. Activation of cumulus cell SMAD2/3 and epidermal growth factor receptor pathways are involved in porcine oocyte–cumulus cell expansion and steroidogenesis. Mol Reprod Dev. (2011) 78:391–402. 10.1002/mrd.21312 21520325

[61] Prochazka R Blaha M Němcová L. Significance of epidermal growth factor receptor signaling for acquisition of meiotic and developmental competence in mammalian oocytes. Biol Reprod. (2017) 97:537–49. 10.1093/biolre/iox112 29025011

[62] Richani D Gilchrist BR. The epidermal growth factor network: role in oocyte growth, maturation and developmental competence. Hum Reprod Update. (2018) 24:1–14. 10.1093/humupd/dmx029 29029246

[63] Prochazka R Kalab P Nagyova E. Epidermal growth factor-receptor tyrosine kinase activity regulates expansion of porcine oocyte-cumulus cell complexes *in vitro*. Biol Reprod. (2003) 68:797–803. 10.1095/biolreprod.102.005520 12604628

[64] Shimada M Umehara T Hoshino Y. Roles of epidermal growth factor (EGF)-like factor in the ovulation process. Reprod Med Biol. (2016) 15:201–16. 10.1007/s12522-016-0236-x 29259438 PMC5715866

[65] Reizel Y Elbaz J Dekel N. Sustained activity of the EGF receptor is an absolute requisite for LH-induced oocyte maturation and cumulus expansion. Mol Endocrinol. (2010) 24:402–11. 10.1210/me.2009-0267 20009084 PMC5428124

[66] Fan H-Y Liu Z Shimada M Sterneck E Johnson PF Hedrick SM. MAPK3/1 (ERK1/2) in ovarian granulosa cells are essential for female fertility. Science. (2009) 324:938–41. 10.1126/science.1171396 19443782 PMC2847890

[67] Gan Y Shi C Inge L Hibner M Balducci J Huang Y. Differential roles of ERK and Akt pathways in regulation of EGFR-mediated signaling and motility in prostate cancer cells. Oncogene. (2010) 29:4947–58. 10.1038/onc.2010.240 20562913

[68] Huang S-Z Wei M-N Huang J-R Zhang Z-J Zhang W-J Jiang Q-W. Targeting TF-AKT/ERK-EGFR pathway suppresses the growth of hepatocellular carcinoma. Front Oncol. (2019) 9:150. 10.3389/fonc.2019.00150 30931258 PMC6428933

