# Supplementary figures and images for: Beneficial Effects of Neurotrophin-4 Supplementation During in vitro Maturation of Porcine Cumulus-Oocyte Complexes and Subsequent Embryonic Development After Parthenogenetic Activation
Source: Front Vet Sci. 2021 Nov 12;8:779298. doi: 10.3389/fvets.2021.779298 (PMC8632945; doi:10.3389/fvets.2021.779298)

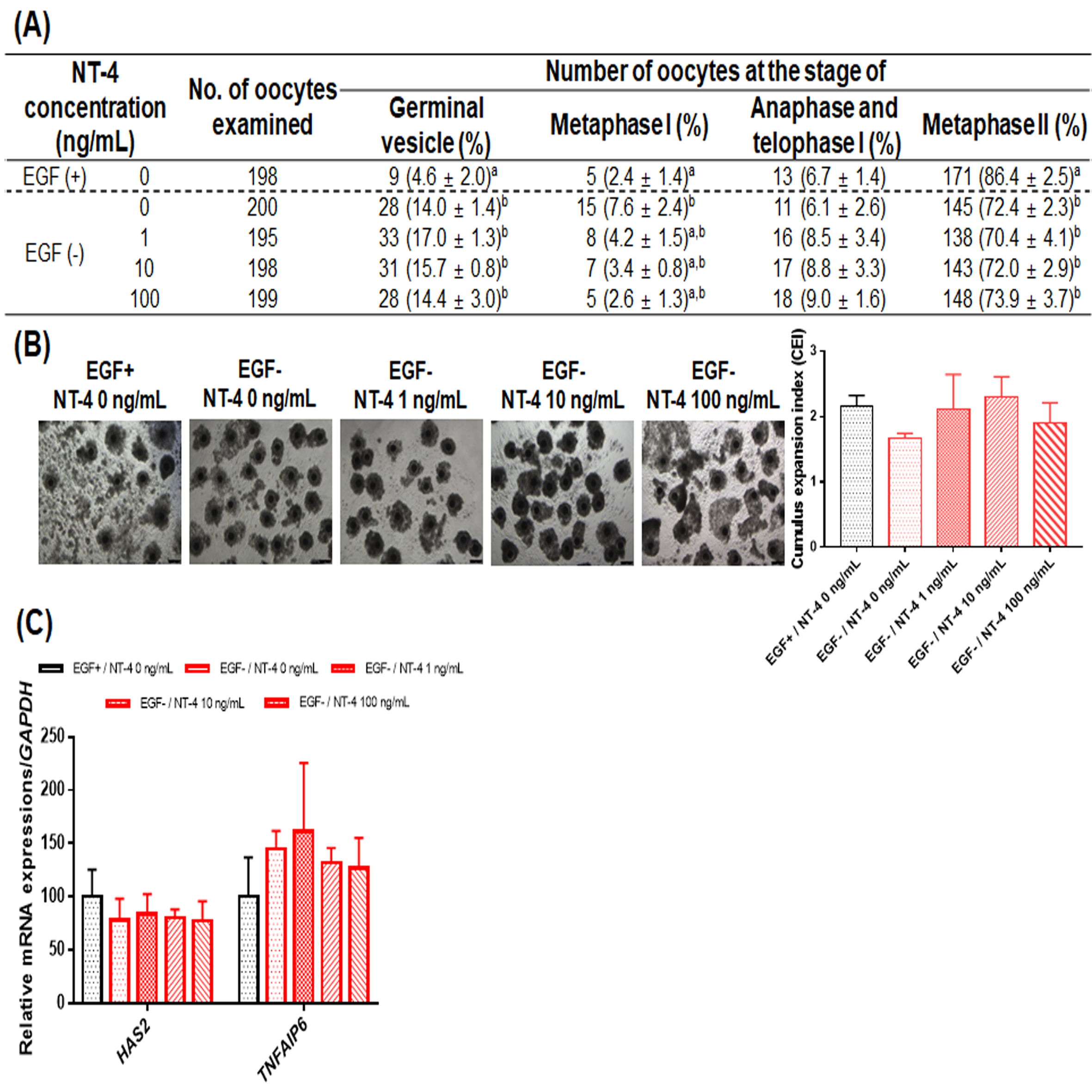

Supplement: Supplementary Figure 1 — Effect of neurotrophin-4 (NT-4) treatment without epidermal growth factor (EGF) during in vitro maturation (IVM) of oocytes and cumulus cell (CC) expansion in porcine cumulus-oocytes complexes (COCs). (A) Evaluation of nuclear maturation rate in porcine COCs treated with NT-4 in the absence of EGF during IVM. a,bValues in the same column with different superscripts significantly differ (p < 0.05). (B) Morphological CC expansion of COCs treated with NT-4 in the absence of EGF during 42 h of IVM. The degree of CC expansion was examined using the cumulus scoring system: 0 (no expansion) to +4 (maximum expansion). Scale bars = 200 μm. (C) Expression of cumulus expansion-related genes (HAS2 and TNFAIP6) in CCs treated with NT-4 in the absence of EGF during 42 h of IVM. The mRNA levels were normalized to GAPDH expression as a control. All data are expressed as the means ± SEM. All experiments were replicated three times. [file Image_1.TIF]
