# Supplementary material for: Beneficial Effects of Neurotrophin-4 Supplementation During in vitro Maturation of Porcine Cumulus-Oocyte Complexes and Subsequent Embryonic Development After Parthenogenetic Activation
Source: Front Vet Sci. 2021 Nov 12;8:779298. doi: 10.3389/fvets.2021.779298 (PMC8632945; doi:10.3389/fvets.2021.779298)
Supplement: Supplementary Table 1 — Primer lists for RT-PCR. [file Table_1.DOCX]

Supplementary table 1. Primer lists for RT-PCR.

| **mRNA** | **Primer sequences** | **Product size**  **(bp)** | **GenBank**  **accession number** |
| --- | --- | --- | --- |
| ***NT-4*** | F: 5’-CGATGGATTCGAATTGACAC-3’  R: 5’-ATCGGAGAGCTGTGATTTTTG-3’ | 196 | NM_001243713 |
| ***trTrkB*** | F: 5’-GTTACCAATCACACGGAGT-3’ | 424 | XM_021064650 |
|  | R: 5’-CATCCAGTGGGATCTTATGAA-3’ |  |  |
| ***TrkB*** | F: 5'-GCTCCATTCATCACAAACAC-3' | 202 | XM_021064647 |
|  | R: 5'-CGGCCTAGAATGCATAGAC-3' |  |  |
| ***p75^NTR^*** | F: 5'-TGGAGATGGAGATGATATGGA-3' | 316 | NM_001244828 |
|  | R: 5'-GGCAATCTCCAATTAGAAGC-3' |  |  |
| ***RN18S*** | F: 5’-CGCGGTTCTATTTTGTTGGT-3’  R: 5’-GGTCATTTCCGACTGAAGAG-3’ | 219 | NR_046261 |

Supplementary table 2. Antibody lists for fluorescent immunohistochemistry.

| **Antibody** | **Host** | **Dilution** | **Cat.** |
| --- | --- | --- | --- |
| **Anti-NT-4** | Mouse | 1:50 | NBP1-47897 |
| **Anti-TrkB** | Mouse | 1:50 | sc-377218 |
| **Anti-Phospho TrkB** | Rabbit | 1:50 | PA5-36695 |
| **Anti-p75^NTR^** | Mouse | 1:50 | 14-9400-82 |
| **Alexa Fluor 488** | Mouse or Rabbit | 1:200 | A11029 (Mouse), A11034 (Rabbit) |
| **Alexa Fluor 594** | Mouse or Rabbit | 1:200 | A11032 (Mouse), A21207 (Rabbit) |

Supplementary table 3. Primer lists for qRT-PCR.

| **mRNA** | **Primer sequences** | **Product size**  **(bp)** | **GenBank**  **accession number** |
| --- | --- | --- | --- |
| ***HAS2*** | F: 5'-TTACAATCCTCCTGGGTGGT-3'  R: 5’-TCAAGCACCATGTCGTACTG-3’ | 199 | NM_214053 |
| ***TNFAIP6*** | F: 5’-TCATAACTCCATATGGCTTGAAC-3’  R: 5’-TCTTCGTACTCATTTGGGAAGCC-3’ | 396 | NM_001159607 |
| ***PCNA*** | F: 5’-CCTGTGCAAAAGATGGAGTG-3’  R: 5’-GGAGAGAGTGGAGTGGCTTTT-3’ | 187 | NM_001291925 |
| ***NRF2*** | F: 5’-CCCATTCACAAAAGACAAACATTC-3’  R: 5’-GCTTTTGCCCTTAGCTCATCTC-3’ | 75 | XM_021075133 |
| ***KEAP1*** | F: 5’-AGCTGGGATGCCTCAGTGTT-3  R: 5’-AGGCAAGTTCTCCCAGACATTC-3’ | 100 | NM_001114671 |
| ***BAX*** | F: 5’-TGCCTCAGGATGCATCTACC-3’  R: 5’-AAGTAGAAAAGCGCGACCAC-3’ | 199 | XM_013998624 |
| ***BCL-2*** | F: 5’-AATGACCACCTAGAGCCTTG-3’  R: 5’-GGTCATTTCCGACTGAAGAG-3’ | 182 | NM_214285 |
| ***GSR*** | F: 5’-TGGGCTCTAAGACGTCACTG-3’  R: 5’-TCTATGCCAGCATTCTCCAG-3’ | 106 | XM_003483635 |
| ***SOD1*** | F: 5'-GTGCAGGGCACCATCTACTT-3’  R: 5'-AGTCACATTGCCCAGGTCTC-3' | 222 | NM_001190422 |
| ***EGFR*** | F: 5’-ATCGGTTTAGGCTACTCACG-3’  R: 5’-GCACAAGGCTGTCCTTATTT-3’ | 193 | NM_214007 |
| ***GRB2*** | F: 5'-GACATAGAACAGGTGCCACA-3'  R: 5'-GTTCACAGGGGTGACGTAAT-3' | 195 | NM_001137628 |
| ***MAPK3*** | F: 5'-ATCACAGTGGAGGAAGCACT-3'  R: 5'-GAGGCATCTGTCCAGGTTAG-3' | 202 | XM_021088019 |
| ***MAPK1*** | F: 5'-AGTCCATCGACATCTGGTCT-3’  R: 5'-GAGCTTTGGAGTCAGCATTT-3' | 240 | XM_021088019 |
| ***AKT1*** | F: 5'-CTACAACCAGGACCACGAGA-3'  R: 5'-CTCATACACATCCTGCCACA-3' | 208 | NM_001159776 |
| ***GAPDH*** | F: 5’-GTCGGTTGTGGATCTGACCT-3’  R: 5’-TTGACGAAGTGGTCGTTGAG-3’ | 207 | NM_001206359 |
| ***RN18S*** | F: 5’-CGCGGTTCTATTTTGTTGGT-3’  R: 5’-GGTCATTTCCGACTGAAGAG-3’ | 219 | NR_046261 |
